# Pilot Study of the Association of the *DDAH2* −449G Polymorphism with Asymmetric Dimethylarginine and Hemodynamic Shock in Pediatric Sepsis

**DOI:** 10.1371/journal.pone.0033355

**Published:** 2012-03-12

**Authors:** Scott L. Weiss, Min Yu, Lawrence Jennings, Shannon Haymond, Gang Zhang, Mark S. Wainwright

**Affiliations:** 1 Division of Critical Care, Department of Pediatrics, Children's Memorial Hospital, Northwestern Feinberg School of Medicine, Chicago, Illinois, United States of America; 2 Department of Pathology and Laboratory Medicine, Children's Memorial Hospital, Northwestern Feinberg School of Medicine, Chicago, Illinois, United States of America; 3 Biostatistics Research Core, Children's Memorial Research Center, Chicago Illinois, United States of America; 4 Division of Neurology, Department of Pediatrics, Children's Memorial Hospital, Northwestern Feinberg School of Medicine, Chicago, Illinois, United States of America,; D'or Institute of Research and Education, Brazil

## Abstract

**Background:**

Genetic variability in the regulation of the nitric oxide (NO) pathway may influence hemodynamic changes in pediatric sepsis. We sought to determine whether functional polymorphisms in *DDAH2*, which metabolizes the NO synthase inhibitor asymmetric dimethylarginine (ADMA), are associated with susceptibility to sepsis, plasma ADMA, distinct hemodynamic states, and vasopressor requirements in pediatric septic shock.

**Methodology/Principal Findings:**

In a prospective study, blood and buccal swabs were obtained from 82 patients ≤18 years (29 with severe sepsis/septic shock plus 27 febrile and 26 healthy controls). Plasma ADMA was measured using tandem mass spectrometry. *DDAH2* gene was partially sequenced to determine the −871 6g/7g insertion/deletion and −449G/C single nucleotide polymorphisms. Shock type (“warm” versus “cold”) was characterized by clinical assessment. The −871 7g allele was more common in septic (17%) then febrile (4%) and healthy (8%) patients, though this was not significant after controlling for sex and race (p = 0.96). ADMA did not differ between −871 6g/7g genotypes. While genotype frequencies also did not vary between groups for the −449G/C SNP (p = 0.75), septic patients with at least one −449G allele had lower ADMA (median, IQR 0.36, 0.30–0.41 µmol/L) than patients with the −449CC genotype (0.55, 0.49–0.64 µmol/L, p = 0.008) and exhibited a higher incidence of “cold” shock (45% versus 0%, p = 0.01). However, after controlling for race, the association with shock type became non-significant (p = 0.32). Neither polymorphism was associated with inotrope score or vasoactive infusion duration.

**Conclusions/Significance:**

The −449G polymorphism in the *DDAH2* gene was associated with both low plasma ADMA and an increased likelihood of presenting with “cold” shock in pediatric sepsis, but not with vasopressor requirement. Race, however, was an important confounder. These results support and justify the need for larger studies in racially homogenous populations to further examine whether genotypic differences in NO metabolism contribute to phenotypic variability in sepsis pathophysiology.

## Introduction

Cardiovascular dysfunction is common in sepsis and is the hallmark of septic shock. Classically, patients with septic shock exhibit hyperdynamic cardiac function and low systemic vascular resistance (SVR) resulting in hypotension and decreased organ perfusion [1]. While this “warm” shock state is typical of adults with sepsis, hemodynamic profiles observed in septic children are more variable, with an increased incidence of low cardiac output and elevated SVR, or “cold” shock [2]. Hemodynamic variability influences therapy, such as choice of vasoactive infusion, and outcomes, with low SVR associated with increased mortality in adult septic shock [3] and decreased cardiac output portending worse survival in pediatric sepsis [4].

The nitric oxide (NO) pathway, through its effects on both myocardial and vascular function, influences hemodynamic changes in septic shock. NO acts through cyclic guanosine monophosphate to sequester intracellular calcium, which enhances vascular and myocardial muscle relaxation [5]. In sepsis, evidence supports an overall increase in systemic NO production [6], [7], [8]. However, multiple factors can affect tissue-specific NO signaling, including availability of arginine (the NO synthase [NOS] substrate) [9], location of distinct NOS isoforms [5], and presence of endogenous NOS inhibitors, the most important of which is asymmetric dimethylarginine (ADMA) [10]. Whether genetic variability in the regulation of NOS activity contributes to different hemodynamic states observed in pediatric septic shock is not known.

The importance of ADMA in regulating NO has been increasingly recognized in disorders of endothelial and vascular dysfunction [10]. ADMA is synthesized by the methylation of arginine residues on proteins and released into circulation during catabolism [10]. ADMA inhibits NO production through competitive binding with arginine for all NOS isoforms and for cellular uptake [10]. Elevated plasma concentrations of ADMA are seen in adults with sepsis [11], multi-organ dysfunction [12], and severe malaria [13] and have shown promise as a biomarker in cardiovascular [14], [15] and renal disease [16]. In pediatric sepsis, however, ADMA levels are more variable, with an overall decrease compared with non-septic controls [17].

**Figure 1:**
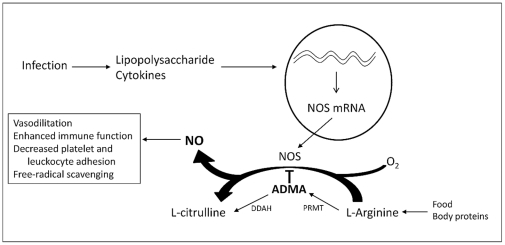
NO-ADMA-DDAH pathway. Onset of infection in sepsis leads to an up-regulation of inducible nitric oxide synthase (NOS) in response to lipopolysaccharide and pro-inflammatory cytokines. This consequently increases nitric oxide (NO) production, which has a multitude of effects on endothelial, vascular, and immune function. Asymmetric dimethylarginine (ADMA) is produced by the methylation of arginine residues on proteins by the enzyme protein-arginine methyltransferase (PRMT) and metabolized by dimethylarginine dimethylaminohydrolase (DDAH). ADMA competitively inhibits NOS, thereby limiting NO production.

Genetic polymorphisms in the enzyme dimethylarginine dimethylaminohydrolase (DDAH), which is responsible for ADMA metabolism (Figure 1), have been associated with different levels of ADMA in adult sepsis [11], diabetes mellitus [18], and pre-eclampsia [19], and co-localization of DDAH with NOS expression supports a key role for this enzyme in the regulation of NO activity [10]. Two distinct isoforms exist, with DDAH1 predominating in the liver and kidneys and DDAH2 primarily found in endothelial, vascular, and immune cells [20]. The predilection for DDAH2 in vascular tissue suggests that variability in expression or activity of this enzyme may contribute to hemodynamic changes observed in sepsis. Polymorphisms in the *DDAH2* gene (OMIM #604744) have been associated with plasma ADMA levels in adult sepsis [11] and vasopressor use following cardiac surgery [21]. We therefore hypothesized that two functional polymorphisms in the promoter region of the *DDAH2* gene—the −871 6g/7g insertion/deletion and −449G/C single nucleotide polymorphisms—affect plasma ADMA concentration and hemodynamic changes in pediatric septic shock.

The objectives of this study were to determine if functional polymorphisms in the *DDAH2* gene were associated with plasma ADMA concentration, distinct hemodynamic states, and cardiovascular dysfunction in pediatric septic shock. We found that the −449G single nucleotide polymorphism (SNP) within the *DDAH2* gene was associated with both decreased plasma ADMA and an increased likelihood of presenting with “cold” shock in pediatric sepsis.

## Methods

### Ethics

Ethics approval was obtained from the Institutional Review Board of Children's Memorial Hospital, Chicago, Illinois. Written informed consent and assent were obtained for study enrollment and laboratory analysis from parents/guardians and patients (age ≥12 years), respectively, with specific consent sought for genotyping.

### Objectives

The objectives of this study were to determine if functional polymorphisms within the *DDAH2* gene influenced susceptibility to developing severe sepsis or septic shock and were associated with differences in concentrations of plasma ADMA concentration, type of shock (“warm” versus “cold”), and vasopressor requirement in pediatric patients. We hypothesized that a higher frequency of rare *DDAH2* genotypes would be evident in children with severe sepsis or septic shock compared with non-septic controls, the likelihood for septic children to present with “warm” versus “cold” shock would differ by *DDAH2* genotype, and genetic variability would be associated with plasma ADMA on PICU admission and longitudinal requirement for pharmacologic hemodynamic support.

### Participants

Patients who were ≤18 years-old and met criteria for severe sepsis or septic shock as defined by the International Pediatric Consensus Conference [22] were recruited from a 42-bed pediatric intensive care unit (PICU) at an academic medical center between May 2009 and June 2010 (septic patients). Exclusion criteria were cardiac arrest preceding admission, treatment with inhaled NO or sildenafil, supplementation with arginine, citrulline, or carnitine, a metabolic, urea cycle, or mitochondrial disorder, chronic renal or hepatic impairment, unrepaired cyanotic heart disease or single-ventricle anatomy, major surgery within the previous 72 hours, transfer from another facility with ongoing sepsis >24 hours, and prior study enrollment. Age-matched control patients from the same hospital were enrolled into two groups: 1) febrile (temperature ≥38.5°C) patients evaluated for infection without severe sepsis or shock (febrile controls) and 2) afebrile patients without evidence of an active infectious or inflammatory condition undergoing a minor procedure, such as a hernia repair or endoscopy (healthy controls). Only those disorders also listed for septic patients (e.g. chronic renal impairment) were used to exclude controls.

### Data Collection

Septic patients were enrolled within 24 hours of PICU admission or, if already in the PICU, within 24 hours of onset of severe sepsis or septic shock as defined by initial fever and first evidence of organ dysfunction. Clinical data were abstracted from the medical record onto a standardized case report form. Information was collected on age, sex, race/ethnicity, and severity of illness indices including Pediatric Index of Mortality (PIM)-2 [23] and Pediatric Logistic Organ Dysfunction (PELOD) scores [24]. For septic patients, intensity of vasoactive infusion therapy was determined by calculating the daily maximum inotrope score (IS) [25] for the first three study days and duration was measured by the number of vasoactive infusion-free days out of 28. Type of shock (“warm” versus “cold”) was categorized based on the initial clinical assessment of septic patients as documented in the medical record (Table 1) [26].

**Table 1:** Hemodynamic Shock Types.

	Warm Shock	Cold Shock
Pulse pressure	Wide (≥30 mm Hg)	Narrow (<30 mm Hg)
Diastolic blood pressure	Decreased	Normal or Increased
Distal pulses	Bounding	Absent or Weak
Capillary refill	“Flash” or ≤2 seconds	“Delayed” or >2 seconds
Extremity temperature	Warm	Cool

### Measurement of Plasma ADMA

Blood was collected at the time of study enrollment (day 1) for septic patients and controls and on day 3 for septic patients. All specimens were processed in the main hospital laboratory with immediate centrifugation and storage of plasma at −70°C for batched analysis. Plasma ADMA was measured by the gold-standard method of high performance liquid chromatography-tandem mass spectrometry (LC-MS/MS) as previously described [27]—except that ADMA-d^7^ was incorporated as the internal standard, sample supernatant was dried to completion and reconstituted in mobile phase, and a Phenomenex Luna Silica column was used.

### DDAH2 Genotyping

**Figure 2:**
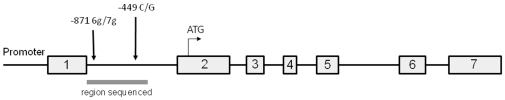
*DDAH2* gene. Schematic representation of *DDAH2* gene (adopted from www.ncbi.nlm.nih.gov/gene/23564), including the upstream promoter, the ATG translation start site, and the −871 6g/7g and −449 G/C polymorphisms. Exons are numbered 1–7. Exon 1 is non-coding, but this area and intron 1 appear to contain a second promoter region.

Buccal swabs were obtained from all patients for genetic analysis. DNA was extracted using the Qiagen EZ1 DNA tissue kit (QIAGEN Inc., Valencia, CA). A 572 base-pair region of the *DDAH2* gene on chromosome 6p21.3 (Figure 2) was then amplified by PCR using Platinum PCR Supermix High Fidelity (Life Technologies Corporation, Carlsbad, CA) for 35 cycles. Sequencing was limited to this region of the *DDAH2* gene because it encompasses an area identified as a second promoter region and includes two polymorphisms that have been previously described to have functional significance—the −871 6g/7g insertion/deletion polymorphism (which lies adjacent to a transcription factor binding site) [28] and the −449 G/C SNP (rs805305) [11], [21], [29], [30]. Denaturation was performed at 96°C for 2 minutes, followed by 5 cycles of 20 seconds at 96°C, 50 seconds at 60°C, and 30 seconds at 72°C, then 30 cycles of 22 seconds at 94°C, 50 seconds at 55°C, and 30 seconds at 72°C, and a final extension at 72°C for 10 minutes. Sequencing was performed using a Big Dye Terminator v1.1 cycle sequencing kit (Life Technologies Corporation, Carlsbad, CA) with M13 forward and M13 reverse primers as sequencing primers (Figure S1), and DNA analysis was performed with 3130xl Genetic Analyzer Data Collection software v3.0 (Life Technologies Corporation, Carlsbad, CA).

### Statistical Methods

Statistical analysis was performed using Statistical Package for the Social Sciences (SPSS Version 12.1, Chicago, IL) and SAS 9.2 (SAS institute Inc., Cary, NC). Since ADMA concentrations, IS, and vasoactive infusion-free days were not normally distributed, results are expressed as the median with interquartile range (IQR) and were compared using the Mann-Whitney *U* and Kruskal-Wallis tests. Deviation from Hardy-Weinberg equilibrium and differences in genotype distributions and type of shock between groups were evaluated using the chi-square and Fisher's exact tests (two-sided). For the −449GC SNP, dominant and recessive models were analyzed for differences in plasma ADMA and shock type. Since population-based studies have reported racial differences in allele frequency for the −449G/C SNP, with the CC genotype observed more commonly in African than European cohorts (http://www.ncbi.nlm.nih.gov/projects/SNP/snp_ref.cgi?rs=805305), logistic regression analysis was used to adjust for sex and race as potential confounders. P-values≤0.05 were considered significant.

## Results

**Figure 3:**
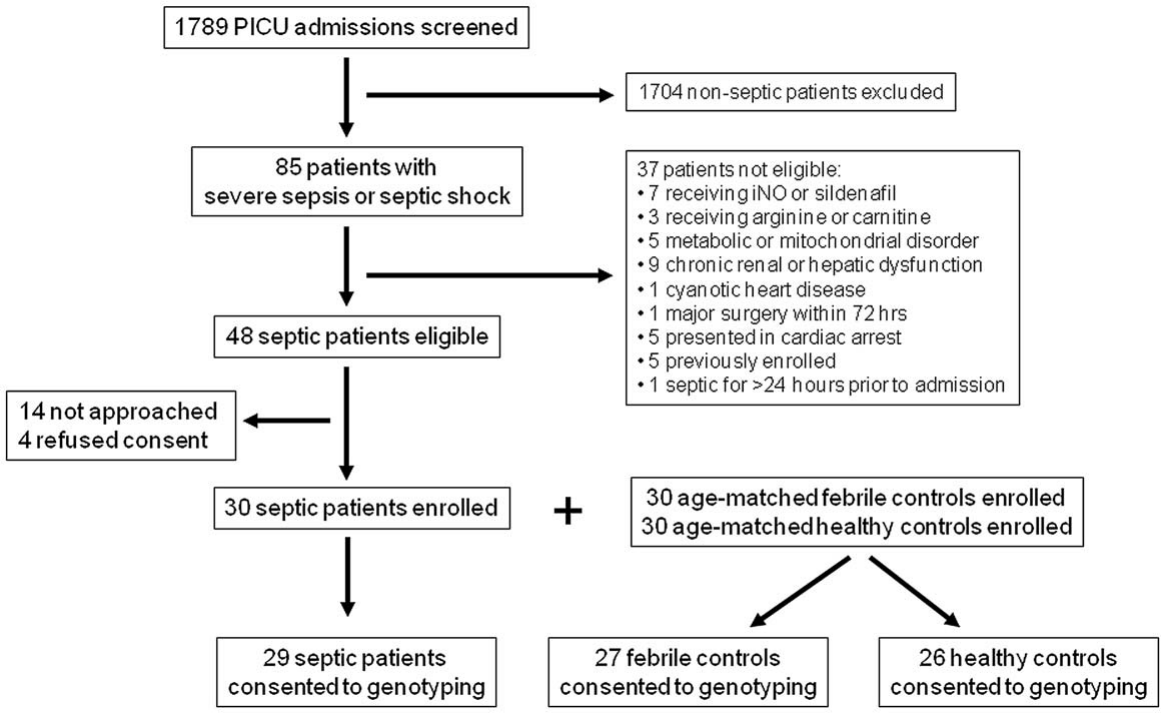
Patient screening and study enrollment. Flow diagram of patient screening and enrollment.

Of the 1789 patients consecutively admitted to the PICU during the study period, 85 (4.8%) met criteria for severe sepsis or septic shock. Thirty-seven of these patients were excluded, with 30 (63%) of the 48 eligible septic patients initially enrolled. Thirty age-matched febrile and 30 age-matched healthy control patients were also initially enrolled. Of these 90 patients, 82 (91%) consented to *DDAH2* genotyping and were included in this analysis (Figure 3). Patient characteristics are shown in Table 2. There was a higher proportion of males in the healthy control group (p = 0.03) and a trend towards a higher proportion of Black and Hispanic patients in the septic and febrile groups (p = 0.08). Bacteremia and pneumonia were the most common sources of infection in septic patients.

**Table 2:** Patient Characteristics.

	Septic	Febrile	Healthy	P value
	n = 29	n = 27	n = 26	
Age (years)	7.4 (2.3–15.4)	10.4 (2.6–16.1)	10.0 (4.3–15.1)	0.83
Sex, % male	48	33	69	0.03
Race/Ethnicity, n (%)				
White, non-Hispanic	9 (31)	7 (26)	17 (65)	0.08
White, Hispanic	13 (45)	13 (48)	5 (19)	
Black	6 (21)	6 (22)	2 (8)	
Other	1 (3)	1 (4)	2 (8)	
PIM-2	6.6 (1.5–9.8)	1.0 (0.8–1.2)	0.8 (0.8–0.9)	<0.001
PELOD	20 (11–21)	0 (0-0)	0 (0-0)	<0.001
Hospital LOS (days)	11 (7–17)	2 (1–4)	1 (1-1)	<0.001
Source of Infection, n (%)				0.007
Bacteremia	12 (42)^a^	0	N/A	
Pneumonia	7 (24)	1 (4)		
Viral syndrome	3 (10)	6 (22)		
Meningitis	0	1 (4)		
Urinary tract infection	0	1 (4)		
No identified source	7 (24)	18 (66)		
Type of Shock, n (%)				
Vasodilated (“warm”)	18 (62)			
Vasoconstricted (“cold”)	9 (31)			
No shock	2 (7)			

N/A = not applicable.

a Gram-positive organism = 4, gram-negative organism = 7, mixed gram-positive and gram-negative organisms = 1.

Observed numbers of each genotype conformed to the Hardy-Weinberg equilibrium. To determine whether the *DDAH2* polymorphisms were associated with susceptibility to severe sepsis/septic shock, we compared genotype distributions between septic and control patients. While there were trends toward an increased frequency of the minor allele −871 7g and −449CC homozygotes in septic patients, overall genotype frequencies did not differ significantly between the three groups (Table 3) or between septic and combined non-septic controls for the −871 6g/7g insertion/deletion polymorphism (p = 0.10) or the −449G/C SNP (p = 0.25).

**Table 3:** *DDAH2* Genotype Frequencies.

*DDAH2* Genotype	Septic	Febrile	Healthy	P value^a^
	n = 29	n = 27	n = 26	
−871 6g/7g Insertion/Deletion				
6g/6g	24 (83)	26 (96)	24 (92)	0.96
6g/7g	5 (17)	1 (4)	2 (8)	
−449G/C SNP				
GG	10 (34)	8 (30)	7 (27)	0.75
GC	10 (34)	13 (48)	15 (58)	
CC	9 (32)	6 (22)	4 (15)	

a Controlled for sex and race.

**Figure 4:**
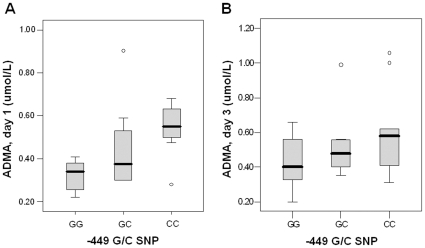
Relationship between plasma ADMA concentration and the *DDAH2* −449G/C genotype in septic patients. Plasma ADMA concentrations differed according to −449G/C genotype on day 1 but not day 3. ADMA was highest in septic patients with −449CC homozygous genotype, intermediate in −449GC heterozygotes, and lowest in the GG homozygotes on day 1 (p = 0.01, panel A). While a similar trend in plasma ADMA was observed on day 3, the difference was not significant (p = 0.33, panel B).

Consistent with the results from all 90 patients [17], the median plasma ADMA on day 1 in the subset in this study was lower in septic patients (0.39, IQR 0.30–0.56 µmol/L) compared with febrile (0.44, IQR 0.40–0.52 µmol/L) and healthy (0.57, IQR 0.53–0.65) controls (p<0.001). There was no difference in plasma ADMA on day 1 or 3 between septic patients with the −871 6g/6g or 6g/7g genotypes (Table 4). For the −449G/C SNP, however, decreasing concentrations of ADMA were observed between genotypes for the septic patients, with the highest levels in CC homozygotes (0.55, IQR 0.49–0.64 µmol/L), intermediate levels in GC heterozygotes (0.37, IQR 0.30–0.56 µmol/L), and lowest levels in GG homozygotes (0.34, IQR 0.25–0.39 µmol/L) on day 1 (p = 0.01) but not day 3 (p = 0.33; Figure 4). In the septic patients with at least one G allele at the −449 position (G-dominant model), plasma ADMA was significantly lower compared to those with the −449CC genotype on day 1 but not day 3 (Table 4). When a G-recessive model was assumed, median ADMA was also lower on day 1 in those with the −449GG homozygous genotype (0.34, IQR 0.25–0.39 µmol/L) compared with the −449GC/CC genotypes (0.50, IQR 0.34–0.61 µmol/L, p = 0.009) but not on day 3 (data not shown, p = 0.20). For the febrile and healthy controls combined, there were no differences in ADMA between distinct −871 6g/7g (p = 0.24) or −449G/C (p = 0.11) genotypes.

**Table 4:** ADMA, type of shock, and vasoactive infusion requirements in septic patients^a^.

	−871 6g/6g	−871 6g/7g	P value
	n = 24	n = 5	
ADMA, day 1 (µmol/L)	0.38 (0.30–0.56)	0.50 (0.34–0.59)	0.49
ADMA, day 3 (µmol/L)	0.47 (0.36–0.60)	0.45 (0.41–0.60)	0.71
Type of Shock, n (%)			
Warm Shock	15 (63)	3 (60)	0.42
Cold Shock	8 (33)	1 (20)	
None	1 (4)	1 (20)	
Inotrope score (day 1)^b^	11 (10–18)	10 (5–17)	0.76
Inotrope score (day 3)^b^	6 (0–14)	6 (2–23)	0.56
Vasoactive infusion-free days	25 (21–26)	25 (23–27)	0.80

	−449 G/G or G/C	−449 CC	P value
	n = 20	n = 9	
ADMA, day 1 (µmol/L)	0.36 (0.30–0.41)	0.55 (0.49–0.64)	0.008
ADMA, day 3 (µmol/L)	0.45 (0.36–0.56)	0.58 (0.39–0.81)	0.20
Type of Shock, n (%)			
Warm Shock	11 (55)	7 (78)	0.01
Cold Shock	9 (45)	0	
None	0	2 (22)	
Inotrope score (day 1)^b^	10 (10–16)	13 (5–18)	0.98
Inotrope score (day 3)^b^	6 (0–14)	0 (0–23)	0.67
Vasoactive infusion-free days	25 (22–26)	26 (22–27)	0.30

a Data are presented as median (IQR), unless indicated.

b Inotrope score = dopamine+dobutamine+(nor-/epinephrine×100)+(milrinone×10)+(vasopressin×10)^25^.

Overall, 18 (62%) of the septic patients presented with “warm” shock and nine (31%) with “cold” shock. The remaining two patients met criteria for severe sepsis but not shock. For the −449G/C SNP, there was a significant difference in genotype frequencies between septic patients with “warm”, “cold”, and no shock when a G-dominant model was assumed (p = 0.01), but not with a G-recessive model (p = 0.48). Nine of the 20 patients (45%) with at least one G allele at the −449 position presented with “cold” shock compared to none with the −449CC homozygous genotype (Table 4). However, significant racial differences were observed in the distribution of −449G/C polymorphisms, with 64% of all Black patients having the CC genotype compared with 14% of all White and Hispanic patients (p<0.001). When race was controlled using logistic regression, the association of shock type with the −449G/C SNP in the septic patients became non-significant (p = 0.32). There was no association between type of shock and different −871 6g/7g genotypes, nor were differences noted for IS on days 1 or 3 or in the number of vasoactive infusion-free days for septic patients with different −871 6g/7g or −449G/C genotypes (Table 4).

**Figure 5:**
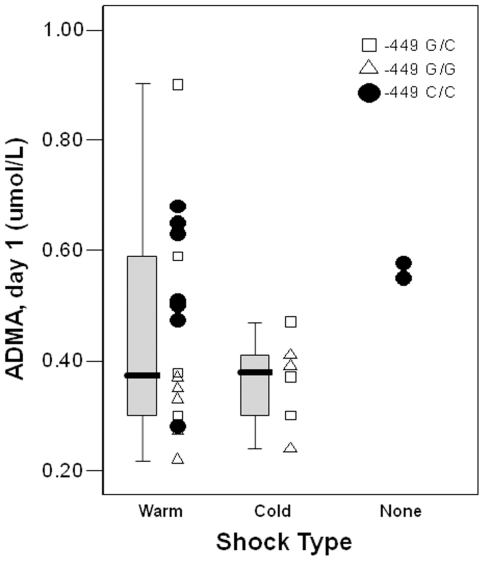
Plasma ADMA and shock type. All of the septic shock patients with the −449CC homozygous genotype exhibited “warm” shock. While day 1 plasma ADMA concentration did not differ between “warm” and “cold” shock (p = 0.42), when compared with −449CC homozygous patients with shock, ADMA was significantly lower in those with the −449G allele for “cold” shock (p = 0.02) and trended lower for “warm” shock (p = 0.09).

Although there was no difference in plasma ADMA level between septic patients with warm or cold shock overall (p = 0.42), ADMA remained lower in the septic patients with at least one −449G allele compared with −449CC homozygotes regardless of type of shock (Figure 5).

## Discussion

This study found that a specific genetic polymorphism—the presence of at least one G allele at the −449 position—within the *DDAH2* gene was associated with low plasma ADMA concentrations in children with severe sepsis or septic shock but not in non-septic controls. This same polymorphism was also associated with an increased likelihood of presenting with vasoconstricted or “cold” shock, with nearly half exhibiting this clinical phenotype compared with exclusively “warm” or no shock seen in the patients with the −449CC homozygous genotype. To our knowledge, this pilot study is the first to associate genetic variability in the *DDAH2* gene with different hemodynamic profiles observed clinically in pediatric sepsis. Our results suggest that genotypic differences in the regulation of NO metabolism may contribute to phenotypic variability in sepsis pathophysiology.

Marked hemodynamic variability occurs in pediatric septic shock. In one study using pulmonary artery catheter measurements in children with fluid-refractory septic shock, only 20% exhibited the classic high cardiac output-low SVR (“warm”) shock state, while 58% had low cardiac output-high SVR (“cold”) shock. Similar findings were reported in a more recent study, with the majority of children with community-acquired sepsis exhibiting “cold” shock [31]. Reflecting these clinical observations, current guidelines recommend a stratified therapeutic approach in pediatric sepsis with vasopressors utilized for persistent “warm” shock and inotropes with or without vasodilators for “cold” shock [26]. The factors underlying individual differences in shock states in sepsis, however, remain unknown.

Alterations in NO bioavailability affect both myocardial and vascular function and therefore directly impact the adequacy of microvascular blood flow, tissue perfusion, and organ function [5]. ADMA decreases NO production through competitive inhibition of all NOS isoforms and intracellular arginine transport [10], and differences in circulating ADMA concentrations have been associated with risk for development of essential hypertension, atherosclerosis, and pulmonary vascular disease [32]. In adults with sepsis, several studies have found elevated plasma ADMA levels [12], [33], particularly in those with septic shock requiring vasoactive infusions [11], [34], suggesting that alterations in ADMA may also have important hemodynamic effects in critical illness. However, in pediatric sepsis, where “cold” shock states are more common, we previously found plasma ADMA to be decreased but did not associate ADMA with shock type in that study [17].

The two DDAH enzymes are responsible for 80–90% of ADMA metabolism and provide an important regulatory mechanism for NO synthesis, and therefore, NO bioavailability [35]. Prior studies have shown that changes in DDAH activity cause alterations in intracellular ADMA in concentrations sufficient to impact NO synthesis [36], [37]. The DDAH2 isoform is found predominantly in endothelial and immune cells, where it co-localizes with endothelial and inducible NOS, respectively. Pharmacologic inhibition of DDAH increases ADMA, which leads to endothelium-dependent vasoconstriction [20], [37] and alters the behavior of circulating lymphocytes [38], supporting the importance of the ADMA-DDAH pathway in regulating NO-mediated vascular and immune function. The identification of genetic polymorphisms that influence expression or activity of the DDAH2 enzyme is therefore intriguing as factors that could affect immune response, susceptibility to infection, and hemodynamic changes that may ultimately impact microvascular blood flow and organ perfusion in pediatric sepsis.

We focused on two polymorphisms within the promoter region of the *DDAH2* gene with previously described functional significance. The −871 6g/7g insertion/deletion polymorphism has been shown to alter the expression and activity of the DDAH2 enzyme *in vitro* [28] and the −449G/C SNP has been associated with alterations in plasma ADMA [11] and clinical outcomes [21], [29]. Despite the potential influence on immune function, neither polymorphism was associated with an increased susceptibility to severe sepsis or septic shock in our study, although we did find a higher percentage of patients with the rare −871 7g allele in the septic group. Jones et al. observed that this −871 7g allele led to an increase in basal *DDAH2* expression in cultured human umbilical vein endothelial cells compared with the −871 6g variant [28]. In contrast, we did not observe a difference in plasma ADMA, as a surrogate measure of DDAH2 activity, between −871 6g/7g genotypes, although the rare occurrence of the 7g allele may have limited our analysis. The −449G/C SNP was evaluated in a previous study of 47 adult patients with severe sepsis or septic shock and carriage of the −449G allele was associated with an increased serum ADMA concentration [11]. These findings contrast with our study, in which we found the −449G allele associated with lower ADMA levels. These conflicting results may reflect that the DDAH2 isoform contributes little to circulating ADMA, as it is more likely that DDAH1 regulates systemic ADMA metabolism [39], [40]. Alternatively, as DDAH2 activity is influenced by multiple factors other than genetics, including availability of L-arginine [41], inflammatory cytokines [10], and negative feedback by NO itself [42], the association of *DDAH2* genotype with plasma ADMA may be influenced by different factors in distinct populations.

While we did not find a relationship between plasma ADMA and type of shock, patients with at least one −449G allele were more likely to exhibit “cold” shock compared with CC homozygotes. Given that the anticipated outcome of ADMA-induced inhibition of NO synthesis should be to oppose vascular relaxation, it is reasonable to expect that a polymorphism associated with decreased plasma ADMA should predispose to increased NO availability, vasodilatation, and “warm” shock. Since patients with the −449G allele exhibited lower plasma ADMA levels irrespective of shock type (Figure 5), the association between this polymorphism and low ADMA may be more of an epiphenomenon and *DDAH2* genotype may influence hemodynamics through a mechanism other than circulating ADMA. The disassociation between *DDAH2* genotype, plasma ADMA, and hemodynamic phenotype is supported by previous studies. Wang et al. reported no difference in serum ADMA with *DDAH2* silencing, but still observed reduced NO-regulated vascular changes [39]. Maas et al. found an increased risk for hypertension in patients with the −449 GG genotype despite no association with plasma ADMA [30]. The −449G allele was also shown to be less common than the CC homozygous genotype in adult patients needing vasopressor support following cardiac surgery [21], and although ADMA levels were not measured, the increased prevalence of the −449G allele in patients with a higher SVR in that study is consistent with our finding that patients with at least one −449G allele were more likely to exhibit “cold”, or high-SVR, shock.

Limitations of our study include reliance on clinical assessment to differentiate “warm” versus “cold” shock rather than direct hemodynamic measurements of cardiac output and SVR and lack of standardization of vasoactive therapy. Since patients may evolve from a warm to a cold shock state (or vice versa), either through natural progression of the septic response or as a consequence of pharmacological intervention, we chose to focus on the association of *DDAH2* polymorphisms and shock state at initial presentation to minimize the influence of vasoactive therapy. However, differences in timing of presentation after onset of infection may itself have influenced hemodynamic state at PICU admission. The association of *DDAH2* polymorphisms with hemodynamic changes after precise initiation of a septic insult and the natural evolution of the septic response in the absence of pharmacologic intervention would be best studied in animal models. Furthermore, we did not differentiate between use of inotrope, vasopressor, and vasodilator agents, which may have contributed to the lack of association between *DDAH2* genotype and vasoactive infusion requirements.

We also recognize that the most important limitation of our study is the small sample size, which increases the risk for spurious conclusions about the association of genetic polymorphisms with clinical variables. To address this concern, three important questions need to be answered [43]: Are the studied populations homogeneous? Does the polymorphism of the gene under study cause a relevant alteration in the level or function of the gene product? Does the product of the studied gene play an important role in the pathogenesis of the disease? The study cohort was derived from an investigation of arginine and ADMA in a heterogeneous population of septic children with controls matched only for age [17]. Given the association of Black race with both the −449CC genotype and “warm” shock, we are not able to differentiate whether the −449G/C SNP itself influences type of shock or is simply a marker for an alternative genetic or environmental difference between racial groups. Further studies in larger, more homogeneous populations are necessary. Second, although the −871 6g/7g polymorphism has been shown to directly affect enzyme expression *in vitro* and the −449G/C SNP has been associated with variable ADMA concentrations in clinical studies, it is not known to what extent genetic differences directly alter expression or activity of the DDAH2 enzyme *in vivo* compared with other regulatory mechanisms, such as oxidative stress, arginine availability, and NO-induced inhibition. Finally, while increasing evidence supports the importance of DDAH in the regulation of NO bioactivity through ADMA metabolism in cardiovascular disease [20], [44], the direct effects of DDAH2-induced changes in intracellular ADMA concentration on NO bioactivity and outcomes in sepsis has not been adequately studied. Therefore, we present this pilot study as a first step, exploratory analysis of a biologically plausible mechanism linking genetic differences to variability in hemodynamic shock in pediatric sepsis, a clinically observed phenomenon which remains poorly understood.

In conclusion, we studied whether two known functional polymorphisms in the *DDAH2* gene are associated with plasma ADMA concentration, distinct hemodynamic states, and cardiovascular dysfunction in pediatric septic shock. We found that the −449G SNP was associated with both decreased plasma ADMA and an increased likelihood of presenting with “cold” shock in pediatric sepsis. Although racial differences emerged as an important confounder that mitigated the association of genotype with shock type, these results support and justify the need to study *DDAH2* polymorphisms in larger, more homogeneous cohorts to examine whether genotypic differences in NO metabolism contribute to phenotypic variability in sepsis pathophysiology. An improved understanding of individual differences in NO metabolism could help to better target therapeutic interventions to critically ill children with hemodynamic compromise.

## Supporting Information

Figure S1 **Primer sequences.** Sequencing was performed using a Big Dye Terminator v1.1 cycle sequencing kit (Life Technologies Corporation, Carlsbad, CA) with M13 forward and M13 reverse primers as sequencing primers.
(DOC)
