# Supplementary material for: Pilot Study of the Association of the DDAH2 −449G Polymorphism with Asymmetric Dimethylarginine and Hemodynamic Shock in Pediatric Sepsis
Source: PLoS One. 2012 Mar 12;7(3):e33355. doi: 10.1371/journal.pone.0033355 (PMC3299781; doi:10.1371/journal.pone.0033355)
Supplement: Figure S1 — Primer sequences. Sequencing was performed using a Big Dye Terminator v1.1 cycle sequencing kit (Life Technologies Corporation, Carlsbad, CA) with M13 forward and M13 reverse primers as sequencing primers. (DOC) [file pone.0033355.s001.doc]

**SUPPORTING INFORMATION FILES**

**Figure S1: Primer sequences**

*DDAH2* forward PCR primer with M13F sequencing tail (shaded region):

5’- TGTAAAACGACGGCCAGTGAGATCACAGCGCGACAG – 3’

*DDAH2* reverse PCR primer with M13R sequencing tail (shaded region):

5’- CAGGAAACAGCTATGACCCCACTAGGATGCGCTCAC – 3’
